# *N*-glycolyl chondroitin synthesis using metabolically engineered *E*. *coli*

**DOI:** 10.1186/s13568-020-01084-6

**Published:** 2020-08-17

**Authors:** Adeola E. Awofiranye, Sultan N. Baytas, Ke Xia, Abinaya Badri, Wenqin He, Ajit Varki, Mattheos Koffas, Robert J. Linhardt

**Affiliations:** 1 Department of Chemical and Biological Engineering, Center for Biotechnology and Interdisciplinary Studies, Rensselaer Polytechnic Institute, Troy, NY USA; 2 Department of Chemistry, Chemical Biology, Center for Biotechnology and Interdisciplinary Studies, Rensselaer Polytechnic Institute, Troy, NY USA; 3 Department of Biological Sciences, Center for Biotechnology and Interdisciplinary Studies, Rensselaer Polytechnic Institute, Troy, NY USA; 4 Department of Pharmaceutical Chemistry, Faculty of Pharmacy, Gazi University, Ankara, Turkey; 5 Glycobiology Research and Training Center, University of California, San Diego, CA USA

**Keywords:** Sialic acid, Biotransformation, *N*-glycolyl chondroitin, Metabolite, *N*-glycolyl glucosamine

## Abstract

*N*-glycolyl chondroitin (Gc-CN) is a metabolite of *N***-**glycolylneuraminic acid (Neu5Gc), a sialic acid that is commonly found in mammals, but not humans. Humans can incorporate exogenous Neu5Gc into their tissues from eating red meat. Neu5Gc cannot be biosynthesized by humans due to an evolutionary mutation and has been implicated in causing inflammation causing human diseases, such as cancer. The study Neu5Gc is important in evolutionary biology and the development of potential cancer biomarkers. Unfortunately, there are several limitations to detecting Neu5Gc. The elimination of Neu5Gc involves a degradative pathway leading to the incorporation of *N*-glycolyl groups into glycosaminoglycans (GAGs), such as Gc-CN. Gc-CN has been found in humans and in animals including mice, lamb and chimpanzees. Here, we present the biosynthesis of Gc-CN in bacteria by feeding chemically synthesized *N*-glycolylglucosamine to *Escherichia coli*. A metabolically engineered strain of *E. coli* K4, fed with glucose supplemented with GlcNGc, converted it to *N*-glycolylgalactosamine (GalNGc) that could then be utilized as a substrate in the chondroitin biosynthetic pathway. The final product, Gc-CN was converted to disaccharides using chondroitin lyase ABC and analyzed by liquid chromatography–tandem mass spectrometry with multiple reaction monitoring detection. This analysis showed the incorporation of GalNGc into the backbone of the chondroitin oligosaccharide.

## Key points

1. *N*-glycolyl chondroitin (Gc-CN) is a stable metabolite of Neu5Gc.
2. Metabolic engineering of *E. coli* K4.
3. *E. coli* has promiscuous enzymes involved in CN biosynthesis.
4. Feeding *E. coli* K4 with GlcNGc affords Gc-CN.

## Introduction

**Fig. 1:**
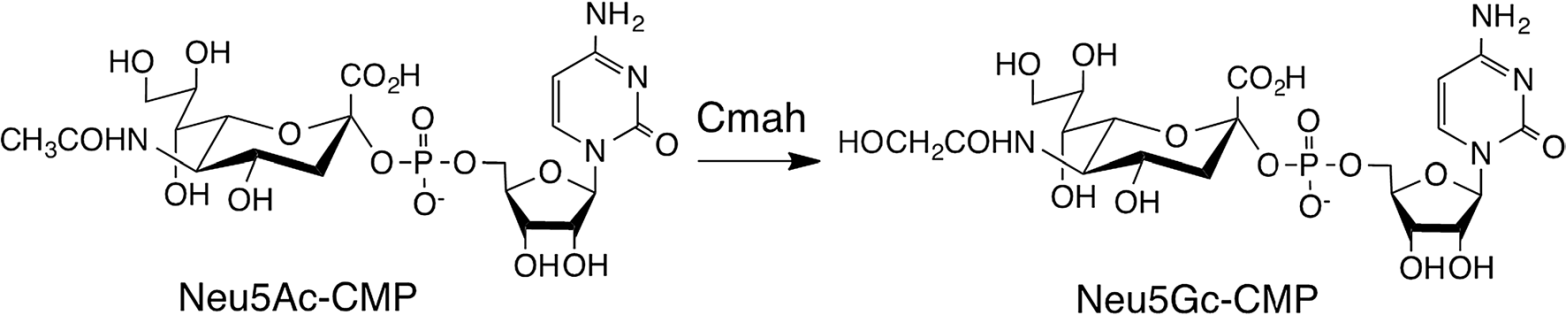
The Cmah enzyme catalyzes the conversion of CMP-Neu5Ac to CMP-Neu5Gc through the addition of an oxygen atom

Sialic acids constitute a family of acidic sugars with a 9-carbon backbone found at the terminal end of glycan chains attached to many soluble glycoproteins (Schauer 2000; Wang and Brand-Miller 2003). Their presence at the terminal end of sugars gives them an advantage in performing their biological roles, providing structure, serving as a ligand for intrinsic and extrinsic receptors, as a binding site for pathogens and toxins, and in molecular mimicry for host invasion (Varki 2008). Sialic acids are mainly expressed in vertebrates and certain bacteria (Angata and Varki 2002). In mammals, the two most common sialic acids found are *N***-**acetylneuraminic acid (Neu5Ac) and *N***-**glycolylneuraminic acid (Neu5Gc). An enzyme called cytidine monophosphate *N*-acetylneuraminic acid hydroxylase (Cmah), encoded by the *CMAH* gene, is responsible for converting CMP-Neu5Ac to CMP-Neu5Gc through the addition of an oxygen atom (Kozutsumi et al. 1990; Bergfeld et al. 2012) (Fig. 1). A few million years ago, a mutation event that occurred in our last common ancestor with the apes, resulted in humans losing the *CMAH* gene and being unable to convert Neu5Ac to Neu5Gc (Chou et al. 1998, 2002).

However, small amounts of Neu5Gc are metabolically incorporated into human tissues from exogenous dietary sources of Neu5Gc like red meat (Samraj et al. 2015). Exogenous sources include other mammals that still synthesize Neu5Gc. Since the human body does not biosynthesize Neu5Gc, a reaction leading to inflammation is triggered when ingested Neu5Gc is incorporated into tissues. The immune system recognizes Neu5Gc as a foreign molecule (xeno-autoantigen) and produces anti-Neu5Gc antibodies (xeno-autoantibodies) present postnatally due to a commensal bacteria (Taylor et al. 2010). These antibodies recognize these foreign molecules (Padler-Karavani et al. 2013) and triggers inflammation (xenosialitis), which is hypothesized to be a key contributor to diseases associated with red meat consumption (Higashi et al. 1977; Samraj et al. 2014). The accumulation of dietary Neu5Gc can lead to local chronic inflammation largely in epithelial and endothelial tissues, and contribute to human pathologies (Tangvoranuntakul et al. 2003; Soulillou et al. 2020). Some carcinomas that have been shown to possess accumulation of Neu5Gc due to its incorporation into epithelial tissues, leading to cancers like lung, gastric, ovarian, prostate and colorectal cancers (Marquina et al. 1996; Carr et al. 2000; Padler-Karavani et al. 2012). Apart from cancers, Neu5Gc when incorporated into endothelial cell can lead to inflammation and cause diseases such as Kawasaki, atherosclerosis and other cardiovascular diseases (Arita et al. 1982; Padler-Karavani et al. 2013; Fernández-Ruiz 2019; Yehuda and Padler-Karavani 2020; Kawanishi et al. 2019).

Moreover, when *Cmah*^−/−^ mice (human-like Neu5Gc-deficient mice) were fed with dietary Neu5Gc and challenged with anti-Neu5Gc antibodies, they developed inflammation, and prolonged exposure to these conditions led to significantly higher cases of cancer (Samraj et al. 2015).

Despite all these finding, proving this hypothesis connecting dietary Neu5Gc to diseases in humans is difficult due to the limitations in directly studying the trace amount of Neu5Gc incorporated. There is no reliable non-invasive method of detecting Neu5Gc incorporation in humans, and the most commonly used methods for detecting sialic acid involves tissue processing (serum does not contain detectable amounts of Neu5Gc) (Tangvoranuntakul et al. 2003). Using antibodies to detect Neu5Gc is also challenging. For example, the Neu5Gc antibody-binding site accommodates 4–5 monosaccharides along with Neu5Gc making it difficult for antibodies to recognize and bind free Neu5Gc not part of a glycan (Padlan and Kabat 1988; Dhar et al. 2019). Another challenge with the detection of Neu5Gc using antibodies is that while some anti-Neu5Gc monoclonal antibodies specifically bind Neu5Gc containing gangliosides (Samraj et al. 2014), gangliosides do not survive paraffin embedding required for immunohistochemistry. This means that some molecules carrying Neu5Gc might not survive to the point of detection.

**Fig. 2:**
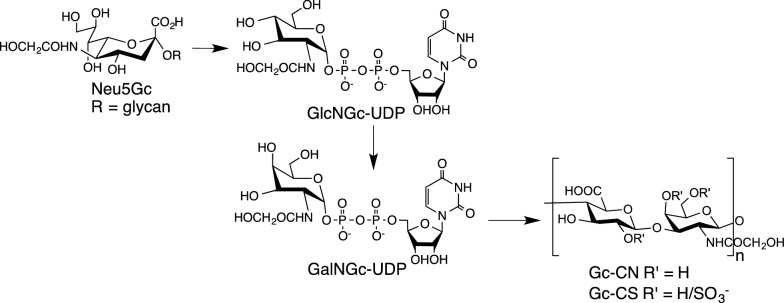
Neu5Gc is converted to UDP-GlcNGc and ultimately UDP-GalNGc. UDP-GalNGc can then be incorporated into Gc-CN and Gc-CS

Human metabolism of Neu5Gc-containing glycans can result in the incorporation of the *N*-glycolyl group into the glycosaminoglycans (GAGs) chondroitin sulfate and unsulfated chondroitin (CN). These GAGs are relatively stable and easy to isolate from serum and analyze making these better candidates for testing the role of dietary Neu5Gc in human diseases. The catabolism of Neu5Gc containing glycans involves six metabolic steps leading to the production of uridine diphosphate *N*-glycolylglucosamine (GlcNGc-UDP) and ultimately uridine diphosphate *N*-glycolylgalactosamine (GalNGc-UDP) (Bergfeld et al. 2017). GalNGc-UDP can then be incorporated into Gc-CN and Gc-CS (Bergfeld et al. 2017) (Fig. 2).

These Gc-CN and Gc-CS derivatives are better suited for analysis because they can be detected from even small volumes of serum and plasma (Lu et al. 2010). Over 95% of GAGs, including CS, can form complexes with plasma proteins (Calatroni et al. 1992) and, therefore, can be isolated from the plasma. Moreover, bikunin is a major CS/CN containing proteoglycan found in both plasma and urine (Chi et al. 2008). A direct method of measuring circulating Gc-CS in these biological samples could serve as an indicator of Neu5Gc incorporation. These samples allow easy detection using reliable analytical methods including high performance liquid chromatography (HPLC) and mass spectrometry (MS), avoiding the need for antibodies (Lu et al. 2010). Gc-CS and its metabolites are very stable as demonstrated by detectable amounts Gc-CS in a 4 million-year-old fossil (Bergfeld et al. 2017).

A microbial source of CN was selected to avoid the risk of sample impurities commonly associated with an animal-derived GAG. Chondroitin sulfate (CS) has been traditionally extracted from animals. Recent efforts to produce CS in microorganism have been successful. CS has been biosynthesized from *E. coli K4*, *Pasteurella multocida*, and *Bacillus megaterium* (Sugiura et al. 2002; He et al. 2015; Jin et al. 2016; Cimini et al. 2018; Restaino et al. 2019). Prior studies have shown that the capsular polysaccharide (CPS) of *E. coli* K4 serotype O5:K4:H4 has a backbone with a repeating disaccharide unit of →4)-β-d glucuronic acid (GlcA) (1→3)-β-d-*N*-acetylgalactosamine (GalNAc), just like chondroitin. However, this CPS also contains an unwanted linked β-fructofuranose branching from the C3 position of GlcA (Rodriguez et al. 1988). The deletion of the *kfoE* gene, responsible for fructosylation, affords an engineered strain that has been optimized for increased chondroitin production (He 2017). In the current study we hypothesized that feeding GlcNGc to *E. coli* could drive the incorporation of *N*-glycolylgalactosamine into chondroitin, through a pathway similar to that involved in *N*-glycolyl incorporation into animal chondroitin sulfate.

## Materials and methods

### Plasmid construction

*E. coli* K4 serotype O5:K4:H4 (U141, 11307) was engineered for the synthesis of chondroitin sulfate. The fructosyltransferase encoded by *kfoE* was deleted using λ red recombinase (Datsenko and Wanner 2000), resulting in strain K4_ΔkfoE. The FRT-flanked kanamycin resistance cassette was PCR amplified from pKD4 by deletion primers with 40 nucleotides homologous regions with a target gene on the genome. The PCR product was purified by a PCR cleanup kit (Cycle Pure Kit, Omega) and transformed into the λ red recombinase expressing *E. coli* K4 strain by electroporation. This system enabled the deletion of the *kfoE* gene and its replacement with an antibiotic resistance gene. Finally, positive knockout strains were screened by colony PCR. Two primers were used in this study. The k4_dkfoE_F primer was 5′ TGCAATATGACCTTAGAAGAGATTTCTAATATGTTAGAACAGGAGAAAAAACACGTCTTGAGCGATTGTG3′. The k4_dkfoe_R primer was 5′ ATATCCAGCCTTGAAAAAACGCGAACTCATCCCCGCCATTGGAATTATAA ACGGCTGACATGGGAATTAG3′.

### Media

Shake flask fermentations utilized rich defined medium developed from modified protocols (Cirino et al. 2006; Neidhardt et al. 1974) (5.0 g/L K_2_HPO_4_, 3.5 g/L KH_2_PO_4_, 3.5 g/L (NH_4_)_2_HPO_4_, 100 mL of 10× MOPS buffer, (83.7 g/L MOPS, 7.2 g/L Tricine, 28 mg/L FeSO_4_·7H_2_O, 29.2 g/L NaCl, 5.1 g/L NH_4_Cl, 1.1 g/L MgCl_2_, 0.5 g/L K_2_SO_4_, 0.2 mL micronutrient stock), 1 mL of 1 m MgSO_4_, 1 mL of 0.5 g/L thiamine HCl, 0.1 mL of 1 m CaCl_2_, 20 g/L glucose, with 12.5 mM GlcNGc. Micronutrient stock consisted of 0.2 g/L (NH_4_)_6_Mo_7_O_24_, 1.2 g/L H_3_BO_3_, 0.1 g/L CuSO_4_, 0.8 g/L MnCl_2_, and 0.1 g/L ZnSO_4_. *E. coli* K4 serotype O5:K4 (L):H4 was from American Type Culture Collection (ATCC 23,502). All reagents for medium preparation were from Sigma Chemical Co. (St. Louis, MO).

### Shake flask experiments

Shake flask experiments were used to evaluate the *N*-glycolyl glucosamine feeding experiments. *E. coli* K4 *ΔkfoE*, cells from 15% glycerol stock were streaked on an agar plate containing 50 µg/mL of kanamycin and grown overnight. Two colonies from the plate were picked for duplicate sample analysis, and pre-cultures were grown overnight at 37 °C. The samples were then diluted to 100 ml at an optical density (OD) 0.05 and transferred to a 250 ml Erlenmeyer flask and incubated at 37 °C with shaking at 220 rpm. The cultures were left to grow under the same conditions for an additional 48 h. The cell growth and chondroitin production was similar to that previously reported in our laboratory (He et al. 2015).

### ***N*-glycolyl chondroitin purification**

CPS was purified from the cell pellet by re-suspending in water and autoclaving in the liquid cycle for 15 min. The autoclaved solution was centrifuged, and the supernatant was collected. Autoclaved supernatant from the cell pellet and cell culture supernatant were precipitated with 80 vol% cold ethanol and stored in an explosion-proof refrigerator overnight at 20 °C. Both intracellular and extracellular chondroitin were recovered. The pellet was collected and re-suspended in buffer (100 mM Tris, pH 7.5, 50 mM MgCl_2_, 10 mM CaCl_2_) and DNAse (1 mg/L, Sigma) was added. The sample was incubated at 37 °C for 1 h after which protease K (2.5 mg/mL, Sigma) was then added, and the sample was incubated at 56 °C for 2 h. After a second precipitation from 80% cold ethanol, the dry pellet was collected, re-dissolved in water (~ 1 mL), and filtered using a 10 KDa spin column (Amicon Ultra, Millipore) to remove small peptides and salt. The chondroitin obtained was sufficiently free of other impurities or other polysaccharides to undertake disaccharide analysis.

### Sample digestion into GAG disaccharides

Digestion buffer (50 mM NH_4_OAc containing 2 mM CaCl_2_ adjusted to pH 7.0) was added to the sample. Recombinant chondroitin lyase ABC (10 mU each, pH optimum 7.4) was added to each sample and mixed. The samples were incubated at 37 °C for 3 days. Under these reaction conditions, chondroitin lyase ABC could depolymerize their GAG substrates (in amounts of over 100 µg) into GAG disaccharides. The samples were washed twice with 100 µL distilled water in 3K MWCO filter unit. The filtrates passing through the filter unit contained disaccharide products, and these were dried using a vacuum centrifuge and stored at − 20 °C for AMAC-labeling.

### AMAC labeling

The dried samples were AMAC-labeled by adding 10 µL of 0.1 m AMAC in DMSO/acetic acid (17/3, V/V) and incubating at room temperature for 10 min, followed by the addition of 10 µL of 1 m aqueous NaBH_3_CN and incubating for 1 h at 45 °C. After the AMAC-labeling reaction, the samples were centrifuged, and each supernatant was recovered. Samples were stored in a light-resistant container at room temperature until analyzed by LC–MS/MS.

### LC–MS/MS analysis

LC was performed on an Agilent 1200 LC system at 45 °C using an Agilent Poroshell 120 ECC18 column (2.7 µm, 3.0 × 50 mm) with a flow rate of 300 µL/min. Mobile phase A (MPA) was 50 mM NH_4_OAc aqueous solution, and the mobile phase B (MPB) was pure methanol. The concentration of MPB increased from 5 to 45% for 10 min, then rose to 100% MPB in the next 0.2 min, and a 4 min flow of 100% MPB was applied to elute all compounds. A triple quadrupole mass spectrometer equipped with an ESI source (Thermo Fisher Scientific, San Jose, CA) was used as a detector. The online MS analysis was at the multiple reaction monitoring (MRM) mode. The conditions and collision energies for all of the disaccharides MRM transitions are listed in our previous publication (Sun et al. 2015). The data analysis was performed on Thermo Xcalibur software.

### ***N*-glycolyl glucosamine (GlcNGc)-synthesis and chemical characterization**

**Fig. 3:**
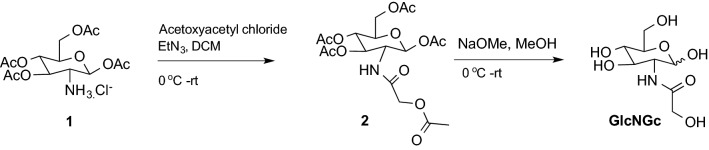
Synthetic route for GlcNGc synthesis

All reagents were purchased from commercial vendors and, unless otherwise noted, used without further purification. “Brine” refers to a saturated aqueous solution of sodium chloride. Thin-layer chromatography (TLC) was performed using Merck Kieselgel 60F254 pre-coated aluminum backed plates. Plates were visualized using 5% H_2_SO_4_ in methanol. NMR experiments were performed on a Bruker Advance III 600 MHz spectrometer (Bruker Bio Spin, Billerica, MA) with Topspin 3.2 software (Bruker). *N*-glycolyl glucosamine (GlcNGc) was synthesized following a previously published strategy (Fig. 3). Briefly, the hydrochloride salt of 2-amino-2-deoxy-1,3,4,6-tetra-*O*-acetyl-β-d-glucopyranose **1** was treated with acetoxyacetyl chloride in the presence of triethylamine to afford acetoxyacetyl residue on the amine group. By applying Zemplen conditions, fully protected intermediate **2** was next deprotected, and GlcNGc was achieved in good yield. Spectral properties of the desired products compounds **2**, and GlcNGc matched those described previously (Sinaÿ 1971; Bergfeld et al. 2012; Macauley et al. 2012).

### 1,3,4,6-Tetra-*O*-acetyl-2-acetyloxyacetamido-2-deoxy-glucopyranose 1

The hydrochloride salt of 2-amino-2-deoxy-1,3,4,6-tetra-*O*-acetyl-β-D-glucopyranose **1** (2.0 g, 5.2 mmol) was dissolved in CH_2_Cl_2_ (20 mL), then triethylamine (2.2 mL, 16 mmol) was added to the solution. The reaction mixture was cooled to 0 °C, and acetoxyacetyl chloride (0.7 mL, 6.5 mmol) was added. The resultant mixture was stirred for 3 h at room temperature. The reaction was monitored by TLC. When the reaction was completed, the mixture was diluted with EtOAc, and the organic phase was washed successively with water, 1 m NaOH, 0.1 m HCl and, brine. The organic phase was dried over MgSO_4_, filtered, and concentrated to yield a white crystalline solid. The material was recrystallized using a mixture of ethyl acetate and hexanes to yield the desired compound **2** as a white solid (1.9 g, 81%). 1 h NMR (600 MHz, CDCl_3_), δ (ppm) 6.18 (1 h, d, *J* = 9.2 Hz), 5.68 (1 h, d, *J* = 8.8 Hz), 5.17–5.10 (2 h, m), 4.52 (1 h, d, *J* = 15.4 Hz), 4.39 (1 h, d, *J* = 15.4 Hz), 4.30 (1 h, ddd, *J* = 9.1 Hz), 4.23 (1 h, dd, *J* = 4.3 Hz, *J* = 12.3 Hz), 4.10 (1 h, dd, *J* = 2.2 Hz), 3.78 (1 h, ddd, *J* = 9.2 Hz), 2.14 (3 h, s), 2.09 (3 h, s), 2.07 (3 h, s), 2.02 (3 h, s), 2.01 (3 h, s). 13C NMR (150 MHz, CDCl3), δ (ppm) 171.3, 170.6, 169.7, 169.6, 169.2, 167.5, 92.4, 73.0, 72.1, 67.6, 62.6, 61.6, 52.7, 20.8, 20.7, 20.5. ESI (m/z): [M−H]^−^ calcd. for C_18_H_24_NO_12_ 446.1298, found m/z 446.1296.

### 2-Deoxy-2-hydroxyacetamido***-***d***-***glucopyranose **GlcNGc**

To a solution of 1,3,4,6-tetra-*O*-acetyl-2-acetyloxyacetamido-2-deoxy-β-d-glucopyranose **2** (1.5 g, 3.35 mmol) in MeOH (50 mL) at 0°C, 0.5 m NaOMe (5 mL) was added dropwise. The solution was stirred 2 h at room temperature. Amberlite IR 120 h^+^ resin was added to neutralize the reaction and was filtered. The filtrate was concentrated under reduced pressure to afford the desired product as a white solid (693 mg, 80%). 1 h NMR (600 MHz, H_2_O) δ (ppm) 5.12 (0.7 h, d, *J* = 3.0 Hz), 4.04 (s, 2 h), 3.86 (1 h, dd, *J* = 3.0 Hz, *J* = 10.2 Hz), 3.79 (1 h, m), 3.75 (1 h, m), 3.72 (1 h, m), 3.42 (1 h, t, *J* = 9.6 Hz), 3.39 (1 h, m). ESI (m/z): [M−H]^−^ calcd. for C_18_H_24_NO_12_ 236.0770, found m/z 236.0772.

## Results

**Fig. 4:**
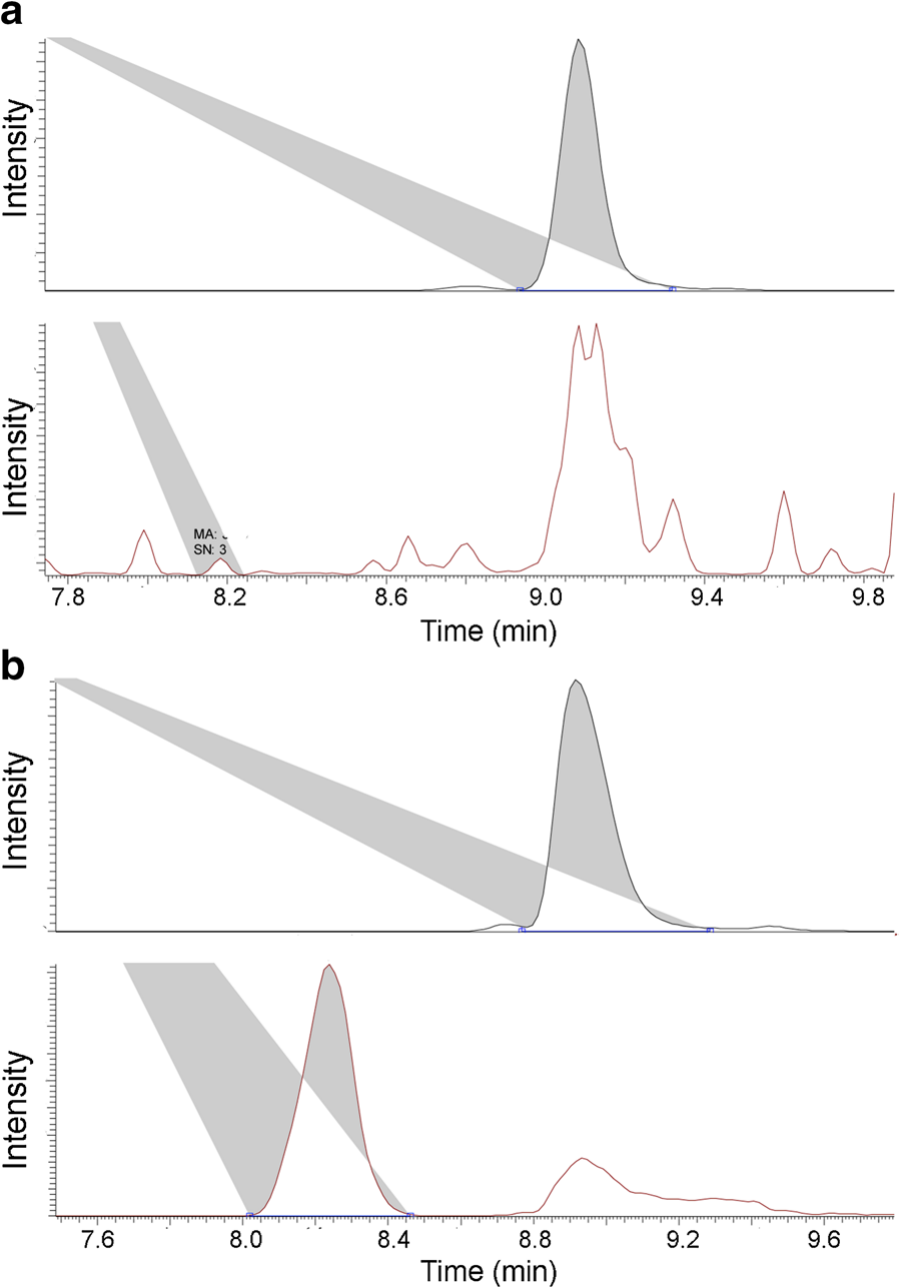
LC–MS/MS MRM result for chondroitin control and *N*-glycolyl chondroitin sample. **a** Chondroitin control has a clear peak (MRM transition pair 572/396) for CN but no peak for Gc-CN (588/412). **b** *N*-glycolyl chondroitin sample both have clear peaks (572/396) for chondroitin and Gc-CN (588/412)

We exhaustively digested chondroitin samples with chondroitin lyase ABC to afford GAG disaccharides and remove any other minor impurities, such as residual lipopolysaccharides. These digests were then purified and AMAC labeled for LC–MS/MS-MRM to verify the presence of Gc-CN. A low capillary temperature and spray voltage was maintained to minimize in-source fragmentation of AMAC-labeled Gc-CN oligosaccharides in MRM (Sun et al. 2015). The 572/396 precursor/product ion pair was used for AMAC-labeled chondroitin disaccharide transition. For AMAC-labeled Gc-CN disaccharide, there is a 16 Da molecular mass increase in the precursor at a mass of 588 used. In the product ion survey, the most sensitive product ion generated had a mass of 412. Therefore, the precursor/product ion pair of 588/412 was used for AMAC-labeled Gc-CN disaccharide transition. From our chondroitin producing construct, serotype O5:K4:H4, only chondroitin was produced as signified by the absence of AMAC-labeled Gc-CN disaccharide (Fig. 4a). From the GlcNGc fed culture, both AMAC-labeled CN and Gc-CN disaccharides were observed at the expected m/z ratio (Fig. 4b). The peak corresponding to the AMAC-labeled Gc-CN disaccharide had an area of 93,312, with a very high signal to noise value of 27,389. MRM unequivocally demonstrated that Gc-CN had been synthesized. Chondroitin and Gc-CN may have different ionization efficiencies, and since there is no Gc-CN standard, it is difficult to calculate the absolute concentration of Gc-CN. Thus, we are unable to estimate the percent conversion of chondroitin to Gc-CN, although based on peak areas it is probably produced at a relatively low level (< 1%).

## Discussion

In this study, we explored the use of metabolically engineered *E. coli* K4 strain, fed with GlcNGc, to produce Gc-CN. The K4 wild type strain was first modified by deleting the *kfoE* gene to prevent the fructosylation of its CPS (He 2017). The resulting organism produces a substantial concentration of chondroitin (~ 300 mg/L) that is both secreted and intracellular. This bacterial strain produces a cell capsule containing unsulfated chondroitin (Rodriguez et al. 1988). The chondroitin present on the cell capsule and within the cell as well as the shed chondroitin can be isolated from the cell pellet and supernatant, respectively. Moreover, studies have shown that chondroitin is present both intracellularly and extracellularly (He et al. 2015). Small-scale shake flask experiments were carried out on this strain with growth on rich, defined media. The results verified the production of chondroitin using LC–MS/MS-MRM. This method required the conversion of chondroitin to the chondroitin disaccharide, ΔUAGalNAc (where ΔUA is a 4-deoxy-*α*-l-*threo*-hex-4-enopyranosyluronic acid), by using chondroitin ABC lyase. The resulting disaccharide was then analyzed by an ultrasensitive and reliable method for disaccharide analysis LC–MS/MS MRM with a limit of detection (LOD) < 1 ng/mL and limit of quantification (LOQ) < 4 ng/mL (Sun et al. 2015; Yu et al. 2019)

**Fig. 5:**
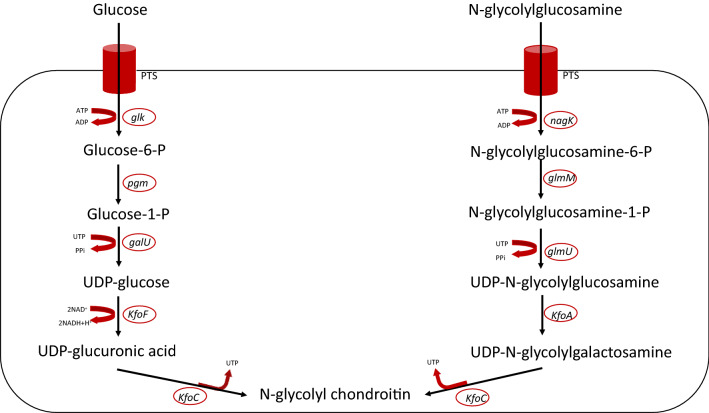
Metabolic pathway for *N*-glycolyl chondroitin synthesis in *E. coli* K4

We next synthesized GlcNGc (Fig. 3), a sugar that we hypothesized would be converted to GalNGc and incorporated in chondroitin during its biosynthesis (Fig. 5) to produce Gc-CN. *E. coli* K4 cells in their CPS biosynthetic pathway make both UDP-GlcA and UDP-GalNAc, the two sugars required to biosynthesize chondroitin. We hypothesized that on feeding GlcNGc engineered *E. coli* K4 would synthesize UDP-GalNGc, and together with UDP-GlcA, would be converted to Gc-CN. As expected, UDP-GalNGc was synthesized using this pathway (Fig. 5), and *E. coli* phosphotransferase system (PTS) was able to transport GlcNGc along with glucose and GlcNAc into the cell. GlcNGc, taken up into the cell through the phosphotransferase system (PTS), was phosphorylated by *N*-acetyl-d-glucosamine kinase (*NagK*) (Uehara and Park 2004) to afford GlcNGc-6-P. Next, phosphoglucosamine mutase (*GlmM*), responsible for the conversion of glucosamine-6‐phosphate to glucosamine‐1‐phosphate (Jolly et al. 1999) catalyzed the conversion of GlcNGc-6-P to GlcNGc-1-P. Next, GlcNGc was converted by the bifunctional enzyme (*GlmU*) (Mengin-Lecreulx and van Heijenoort 1994), to UDP-GlcNGc. UDP-glucose 4-epimerase (*KfoA*) (Zhu et al. 2018) catalyzed the conversion of UDP-GlcNGc to UDP-GalNGc. UDP-GalNGc and UDP-GlcA were then converted to Gc-CN through the action of a chondroitin polymerase, (*KfoC*) (Zanfardino et al. 2010). This pathway demonstrates that all the enzymes in this biosynthetic pathway (Fig. 5) are able to accept the *N*-glycolyl functional group in place of the natural *N*-acetyl functional group, suggesting enzyme promiscuity.

These preliminary studies showed about 1% conversion to Gc-CN. The exact concentration of synthesized Gc-CN could not be determined due to the absence of standards. Comparatively, a Gc-CN could be chemically synthesized in a 10% yield (Bergfeld et al. 2017). There are two likely reasons for the relatively low levels of Gc-CN afforded through fermentation. The first reason is that the cells were fed with a relatively low concentration (12.5 mM) of GlcNGc, providing only a small amount of *N*-glycolyl for incorporation into CN. Future feeding experiments will examine the use of higher concentrations of GlcNGc or per-acetylation to increase conversion of CN to Gc-CN. The second reason is that there may be competition for resources between the production of UDP-GalNGc from GlcNGc (Fig. 5) with the production of UDP-GalNAc from glucose (He et al. 2015; Cimini et al. 2015). *E. coli* may be diverting most of its resources to the familiar and preferred pathway from glucose to UDP-GalNAc. We hypothesize that it may be possible to knock out the bifunctional *glmU* gene to prevent this competition. This gene encodes a bi-functional enzyme responsible for both the uridyltransferase and acetyltransferase activity (Mengin-Lecreulx and van Heijenoort 1994). The acetyltransferase catalyzes the acetylation of glucosamine-1-phosphate (GlcN-1-P), forming *N*-acetylglucosamine-1-phosphate (GlcNAc-1-P) (Mengin-Lecreulx and van Heijenoort 1993) and, the uridyltransferase catalyzes the formation of UDP-*N*-acetylglucosamine (UDP-GlcNAc) (Mengin-Lecreulx and van Heijenoort 1994). In the production of Gc-CN, the acetyltransferase domain is not required. Therefore, it may be possible to mutate the *glmU* gene to inactivate the acetyltransferase while maintaining uridyltransferase activity. Such a mutation should stop the cells from making GalNAc, so that all of the cell’s resources can be diverted to GalNGc synthesis. Modulating this gene could also be useful in generating Gc-CN standards. Such modulation has been reported (Pompeo et al. 1998).

In conclusion, we demonstrate the first production of Gc-CN in an engineered strain of *E. coli.* While the levels of Gc-CN residues are relatively low, they are similar to the Gc-CN content expected on human consumption of red meat. Enhancement of incorporation of GalNGc into chondroitin might be achieved through optimized feeding of GlcNGc, by knocking out enzymes in the biosynthetic pathway or through the protein engineering of bifunctional biosynthetic enzymes. Future studies will examine all three of these approaches.

## Data Availability

All data and materials are available upon request.
